# Nasal spray (Zavegepant) for migraines: a mini-review

**DOI:** 10.1097/MS9.0000000000000843

**Published:** 2023-05-15

**Authors:** Muhammad Omar Larik, Muhammad Ashhal Iftekhar, Bilal Ulhassan Syed, Omema Ansari, Mozaena Ansari

**Affiliations:** a Dow International Medical College, Dow University of Health Sciences; b Sindh Medical College, Jinnah Sindh Medical University, Karachi, Pakistan

**Keywords:** BHV-3500, CGRP antagonist, intranasal, migraine, Zavegepant

## Abstract

Neurological disorders, especially migraines, pose a significant global burden. This has driven the recent innovative research being conducted in the field of anti-migraine therapies, including the discovery of Zavegepant for the treatment of acute migraine attacks. Zavegepant is a novel, first-in-class, intranasally administered calcitonin gene-related peptide (CGRP) receptor antagonist that has recently been approved for use in acute migraine attacks. Recent randomized controlled trials comparing Zavegepant with a placebo have demonstrated favorable results with respect to primary endpoints, as well as a desirable safety profile. The current first-line therapy consists of oral triptans, which are associated with lower efficacy, weaker safety profile, and an unsatisfactory preference rate among patients. Moreover, the intranasal method of administration is a characteristic advantage of Zavegepant, as patients suffering from acute migraine attacks cannot easily ingest oral medication, due to severe nausea and vomiting. In this mini-review, the efficacy and safety of Zavegepant will be compared with those of alternative treatments available for migraines, including oral triptans, intranasal triptans, and other CGRP antagonists currently available in the market. With currently available research, Zavegepant holds superiority over other forms of treatment and can be included in the current treatment guidelines for migraine attacks. However, further research is necessary to effectively assess Zavegepant’s long-term efficacy, safety, tolerability, and drug–drug interactions.

## Introduction

Highlights

- Zavegepant is a novel, first-in-class, intranasally administered, third-generation calcitonin gene-related peptide (CGRP) antagonist for the treatment of acute migraine attacks.
- Recent clinical trials demonstrated impressive efficacy, alongside a favorable safety profile with mild adverse events and minimal severe adverse events.
- In comparison to current first-in-line therapy, oral triptans show lower efficacy and a discouraging safety profile. Zavegepant, on the other hand, excels in all aspects.
- Intranasal administration is useful for migraine patients who are unable to consume medication orally due to severe nausea and/or vomiting.
- Zavegepant has the opportunity to contest against other widely used anti-migraine therapies and possibly replace the current treatment guidelines for acute migraine attacks.

**Figure 1:**
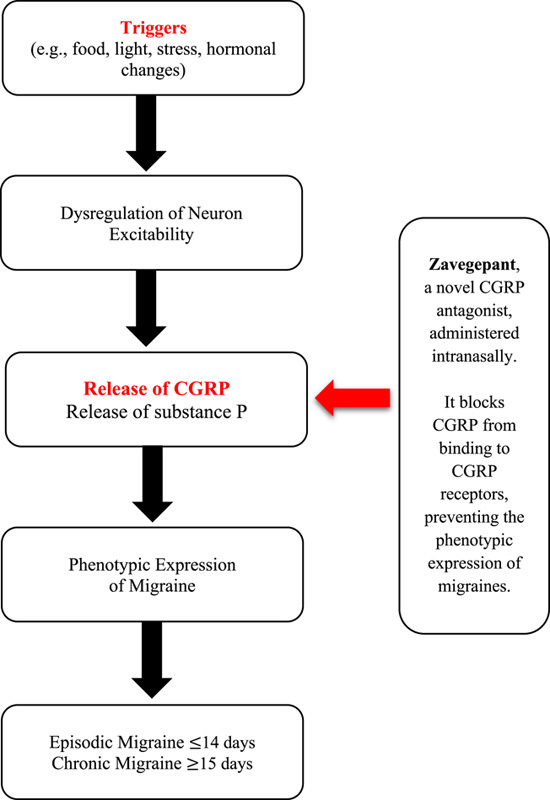
Summary of pathophysiology of migraine. CGRP, calcitonin gene-related peptide.

Migraine is a complex neurobiological disorder with disabling headaches in association with heightened excitability of the central nervous system (CNS), further characterized by recurring attacks^1,2^. An episodic attack may last anywhere from 4 to 72 h, typically characterized by four overlapping phases, including the (i) premonitory phase, (ii) aura, (iii) headache, and (iv) postdrome^3^. Migraines are thought to be induced by certain triggers, such as the commencement of menstruation, stress, tiredness, or exhaustion, and certain foods and drinks^2^. In accordance with the International Classification of Headache Disorders (ICHD), the ‘third edition’ diagnostic criteria for chronic migraine include headache for at least 15 days per month (with migraine-associated features for a minimum of 8 of the days), whereas the diagnostic criteria for episodic migraine involve headache for 14 or less days per month^4,5^. Alternatively, a migraine may be classified as a “migraine with aura”, or a “migraine without aura”^5^. Migraines pose a significant global burden, with the incidence of migraine estimated to be 14 107 per 100 000 in 2019 – an increase of 1.7% since 1990^6^. In high-income countries, the estimated prevalence of active headache disorders is 52%, with the prevalence of migraine at 14%, making migraines a relatively familiar disorder^7^. The inevitable and rising burden of this disease has led to significant innovation in the research of anti-migraine therapies. The summary of the pathophysiology of migraine has been highlighted in Figure 1.

In this mini-review, the efficacy and safety of Zavegepant will be compared with those of alternative treatments available for migraines, including sumatriptan (an oral as well as an intranasally administered triptan), and other calcitonin gene-related peptide (CGRP) antagonists currently available in the market.

## Current treatment guidelines for migraines

Current treatment guidelines for the management of mild-to-moderate migraine headaches consist of the use of simple analgesics (acetaminophen, NSAIDs) or a combination of analgesics, which serve to be much more cost-effective when compared to migraine-specific treatment, which is customarily used in acute episodes^8^. For moderate-to-severe migraine attacks, migraine-specific treatment is the first line of choice, including oral triptans or a combination of sumatriptan and naproxen^9^. Medication for severe, debilitating migraine attacks (status migrainosus) lasting more than 72 h will be treated with intravenous infusions of ketorolac, valproate, or dihydroergotamine^8,10^.

Triptans are serotonin 5-HT1B and 5-HT1D receptor agonists, and a class of compounds that are primarily used for the treatment of migraine attacks. The first-in-class triptan included sumatriptan, with advancing research creating further generations of triptans, including zolmitriptan, rizatriptan, eletriptan, and more^11^. Most triptans are administered enterally; however, sumatriptan is currently offered as an oral medication, in powdered form, or via an intranasal method of administration. Although intranasal sumatriptan shows similar efficacy to oral sumatriptan, it has a limited therapeutic effect^11^.

## A novel CGRP antagonist – Zavegepant

Zavegepant (BHV-3500) is a novel small molecule CGRP receptor antagonist, a first-in-class drug to receive approval for intranasal administration^12^. It consists of a 37-amino-acid neuropeptide that acts on calcitonin-like receptors, blocking the potentiation of the adenylate cyclase pathway. CGRP is secreted from sensory nerves and acts as a potent vasodilator, making it an important modulator in pain pathways. With respect to migraines, CGRP is released from the trigeminal nerve fibers and acts on the pain-producing meningeal blood vessels^12^. This produces the characteristic expression of migraines. On 9 March 2023, the Food and Drug Administration (FDA) granted approval to Zavzpret (Zavegepant), a nasal spray administered for the symptomatic treatment of patients suffering from acute migraine attacks^13^. This novel drug falls under the gepant class of drugs, which also includes other orally administered drugs such as telecagepant, rimegepant, and ubrogepant. Zavegepant is a third-generation gepant, the only intranasally administered drug in the gepant class, which is an advancement from the hepatotoxic initial generations^14^. The particular point of action in the pathophysiological process of migraine has been highlighted in Figure 1.

## Efficacy and safety of Zavegepant

A phase 2/3, double-blind, placebo-controlled, and dose-ranging randomized controlled trial involving 1581 patients conducted in the United States assessed the efficacy and safety of intranasally administered Zavegepant versus placebo^15^. The average age of included participants was 40.8 years, with preventative migraine medication being used by 13.6% of participants, with the female gender (85.5%) dominating the sample. The duration of the study was 11 weeks. This study demonstrates that Zavegepant, especially when administered at 10 or 20 mg doses, produced favorable effects on specified outcomes when compared to a placebo. The primary endpoints included pain freedom at 2 h post-dose (10 mg: 22.5%, *P*=0.01; 20 mg: 23.1%, *P*=0.006) and freedom from the most bothersome symptom (MBS) at 2 h post-dose (10 mg: 41.9%, *P*=0.02; 20 mg: 42.5%, *P*=0.009)^15^. This illustrates that the coprimary endpoints in this study showed that Zavegepant demonstrates significantly favorable results when compared to placebo. On the other hand, the secondary endpoints denoting relief from pain 2 h post-dose (10 mg: 60.6%, *P*=0.04; 20 mg: 61.2%, *P*=0.03), return to normal function 2 h post-dose (10 mg: 34.5%, *P*=0.04; 20 mg: 34.7%, *P*=0.03), and alongside other outcomes, Zavegepant was shown to be efficacious in nature, with significant differences arising when compared with placebo^15^.

Furthermore, a recent phase 3, double-blind, placebo-controlled, multicenter randomized controlled trial with 1405 participants was conducted, exploring the efficacy and safety of Zavegepant versus placebo^16^. The average age of the included participants was 40.9 years, with the female gender (83%) dominating the sample. The primary endpoints of this study (pain freedom 2 h post-dose and freedom from the MBS 2 h post-dose) were observed to significantly favor Zavegepant. Additionally, various secondary endpoints showed significant effectiveness, including pain relief, sustained pain relief, and sustained pain freedom 2 h post-dose, amongst other endpoints^16^. The efficacy and safety findings of this study are consistent with the previously mentioned phase 2/3 study conducted by Croop *et al.*, highlighting the consistency of benefits provided by Zavegepant.

Furthermore, this novel drug has a favorable safety profile, with no signs of hepatotoxicity indicated. Although cohorts receiving the active drug were noted to suffer from greater treatment-related adverse events in the study conducted by Croop *et al*.^15^, the most common adverse events were relatively mild, and included dysgeusia (13.5%), nausea (4.1%), and nasal discomfort (1.3%). A total of five participants had suffered from a severe adverse event, of which two were from the Zavegepant cohort, and were considered unrelated to the treatment^15^. In the trial conducted by Lipton *et al*.^16^, common adverse events included dysgeusia (21%), nasal discomfort (4%), and nausea (3%), with no severe adverse events observed. This shows the consistency in the safety profile between the two studies conducted.

Presently, the long-term effects, drug–drug interactions, and possible use of combination therapies of Zavegepant have not been explored. Although in-vitro studies have shown a low potential for possible drug–drug interactions with Zavegepant^17^.

## Comparison of Zavegepant with alternative treatments

Zavegepant has the capability of becoming the new norm for the treatment of acute migraine attacks. The intranasal route of administration can be incredibly beneficial for patients who struggle with oral consumption of medication due to extreme nausea and vomiting^12,18^. Moreover, the convincing safety profile of Zavegepant, with its uncommonly mild side effects, makes it an attractive alternative for migraine patients. Currently, Zavegepant is the only intranasal drug in the gepant class. However, in the triptan class of drugs, intranasal sumatriptan may be utilized in patients who have difficulty with oral triptan therapy. Intranasal sumatriptan had shown significant improvement in pain relief and headache relief 2 h post-dose. However, the use of intranasal sumatriptan may be limited due to its unfavorable safety profile attributed to the presence of cardiovascular contraindications. In the United States, up to 10% of migraine patients are restricted from the use of sumatriptans due to such contraindications, including patients of hypertension, ischemic heart disease, and coronary artery vasospasms^15^. This illustrates the benefit of using intranasally administered Zavegepant in lieu of intranasally administered sumatriptan, due to its desirable safety profile.

With respect to the current first-line of treatments in the market, consisting of oral triptans (e.g. eletriptan), studies demonstrate an efficacy of 37.9% of pain relief 2 h post-dose^19^. Conversely, Zavegepant (20 mg) displays an efficacy of 61.2% of pain relief 2 h post-dose^15^. The inconsistency of response of oral triptans is exceptionally concerning for use in acute migraine patients, with relief observed at 64% for two out of three attacks, and 33% for three out of three attacks^20^. Zavegepant shows a clear demonstration of greater efficacy with consistent results, as measured across multiple studies.

Contrastingly, oral triptans, such as sumatriptan, are shown to cause greater adverse effects, including gastrointestinal vascular ischemia and infarction, peripheral vascular ischemia, and Raynaud’s syndrome^21^. Moreover, in a meta-analysis conducted by Yang *et al*.^22^, certain triptans (including sumatriptan) were associated with a greater incidence of total adverse events when compared to CGRP antagonists. Furthermore, triptans have prominent cardiovascular contraindications that result in restricted use within up to 10% of the United States population, creating a potential market for alternative drugs for the treatment of acute migraine attacks^15^. Resultingly, oral triptans are associated with a frightful patient dissatisfaction rate of up to 50%^23^. Though the tolerability of Zavegepant has not been directly compared with that of triptans, the pre-existing literature on the safety profile of CGRP antagonists demonstrates favorable results, especially when compared to triptans.

In addition to the triptan class, the gepant class is approved for the treatment of acute migraines. As mentioned previously, Zavegepant is a third-generation CGRP antagonist administered intranasally, as compared to the oral route of administration of other CGRP antagonists or gepants. Though certain CGRP antagonists typically perform extremely well with respect to their efficacy and safety profile, along with greater patient satisfaction (up to three times greater preference for rimegepant compared to prior treatments), the difficulty in consumption of oral or dissolved medication remains^24^.

## Conclusion

In conclusion, intranasally administered Zavegepant has considerable potential to be a promising alternative for patients who generally struggle with oral triptans (due to inability of oral consumption, poor tolerance, and significant side effects), which has resulted in an alarming patient dissatisfaction rate, making it a less favorable form of treatment. Further comprehensive research comparing Zavegepant and oral triptans, or other intranasally administered drugs, is encouraged in order to arrive at an accurate scientific understanding. Additionally, the long-term efficacy, safety, possible combination therapies, and drug–drug interactions of Zavegepant must be determined through extension studies.

## Ethical approval

Not applicable.

## Consent

Not applicable.

## Sources of funding

The authors declare that no funds, grants, or support was received.

## Author contribution

M.O.L.: conceptualization, literature search, writing of original draft, review of final manuscript, and submission of manuscript; M.A.I.: literature search, writing of original draft, and review of final manuscript; B.U.S.: literature search, writing of original draft, and review of final manuscript; O.A.: literature search, writing of original draft, and review of final manuscript; M.A.: literature search, writing of original draft, and review of final manuscript.

## Conflicts of interest disclosure

The authors declare that there are no conflicts of interest.

## Research registration unique identifying number (UIN)

Not applicable.

## Guarantor

Muhammad Omar Larik.

## Data availability statement

Not applicable – data obtained were from the public database.

## Provenance and peer review

Not commissioned, externally peer-reviewed.

## References

[1] Weatherall MW . The diagnosis and treatment of chronic migraine. Ther Adv Chronic Dis 2015;6:115–123. 25954496 10.1177/2040622315579627 PMC4416971

[2] NHS. Migraine. Accessed 11 March 2023. https://www.nhs.uk/conditions/migraine/

[3] Aguilar-Shea AL Membrilla Md JA Diaz-de-Teran J . Migraine review for general practice. Aten Primaria 2022;54:102208. 34798397 10.1016/j.aprim.2021.102208 PMC8605054

[4] Charles A . The pathophysiology of migraine: implications for clinical management. Lancet Neurol 2018;17:174–182. 29229375 10.1016/S1474-4422(17)30435-0

[5] Olesen J . International classification of headache disorders. Lancet Neurol 2018;17:396–397. 29550365 10.1016/S1474-4422(18)30085-1

[6] Safiri S Pourfathi H Eagan A . Global, regional, and national burden of migraine in 204 countries and territories, 1990 to 2019. Pain 2022;163:e293–e309. 34001771 10.1097/j.pain.0000000000002275

[7] Stovner LJ Hagen K Linde M . The global prevalence of headache: an update, with analysis of the influences of methodological factors on prevalence estimates. J Headache Pain 2022;23:34. 35410119 10.1186/s10194-022-01402-2 PMC9004186

[8] UpToDate. Acute treatment of migraine in adults. Accessed 11 March 2023. https://www.uptodate.com/contents/acute-treatment-of-migraine-in-adults

[9] Becker WJ . Acute migraine treatment in adults. Headache 2015;55:778–793. 25877672 10.1111/head.12550

[10] Raskin NH . Repetitive intravenous dihydroergotamine as therapy for intractable migraine. Neurology 1986;36:995–997. 3520384 10.1212/wnl.36.7.995

[11] Tfelt-Hansen P De Vries P Saxena PR . Triptans in migraine: a comparative review of pharmacology, pharmacokinetics and efficacy. Drugs 2000;60:1259–1287. 11152011 10.2165/00003495-200060060-00003

[12] Noor N Angelette A Lawson A . A comprehensive review of zavegepant as abortive treatment for migraine. Health Psychol Res 2022;10:35506. 35774914 10.52965/001c.35506 PMC9239361

[13] Drugs.com. Zavzpret FDA Approval History. Accessed 10 March 2023. https://www.drugs.com/history/zavzpret.html

[14] Scuteri D Tarsitano A Tonin P . Focus on zavegepant: the first intranasal third-generation gepant. Pain Manag 2022;12:879–885. 36189708 10.2217/pmt-2022-0054

[15] Croop R Madonia J Stock DA . Zavegepant nasal spray for the acute treatment of migraine: a phase 2/3 double-blind, randomized, placebo-controlled, dose-ranging trial. Headache 2022;62:1153–1163. 36239038 10.1111/head.14389 PMC9827820

[16] Lipton RB Croop R Stock DA . Safety, tolerability, and efficacy of zavegepant 10 mg nasal spray for the acute treatment of migraine in the USA: a phase 3, double-blind, randomised, placebo-controlled multicentre trial. Lancet Neurol 2023;22:209–217; Erratum in: Lancet Neurol. 10 March 2023; PMID: 36804093. 36804093 10.1016/S1474-4422(22)00517-8

[17] Bertz R Bhardwaj R Donohue MK . Effects of zavegepant and concomitant sumatriptan on blood pressure and pharmacokinetics in healthy adult participants (P-122). Headache 2022;62(S1):108.

[18] Pierce M . Oral triptans and nausea: treatment considerations in migraine. Headache 2013;53(Suppl 1):17–20. 23721286 10.1111/head.12110

[19] Hou M Liu H Li Y . Efficacy of triptans for the treatment of acute migraines: a quantitative comparison based on the dose-effect and time-course characteristics. Eur J Clin Pharmacol 2019;75:1369–1378. 31446449 10.1007/s00228-019-02748-4

[20] Ferrari A Tiraferri I Neri L . Why pharmacokinetic differences among oral triptans have little clinical importance: a comment. J Headache Pain 2011;12:5–12. 20878535 10.1007/s10194-010-0258-4 PMC3072488

[21] Brar Y Hosseini SA Saadabadi A . Sumatriptan. StatPearls Internet. StatPearls Publishing; 2022. 29262214

[22] Yang CP Liang CS Chang CM . Comparison of new pharmacologic agents with triptans for treatment of migraine: a systematic review and meta-analysis. JAMA Netw Open 2021;4:e2128544. 34633423 10.1001/jamanetworkopen.2021.28544 PMC8506232

[23] Bigal M Rapoport A Aurora S . Satisfaction with current migraine therapy: experience from 3 centers in US and Sweden. Headache 2007;47:475–479. 17445096 10.1111/j.1526-4610.2007.00752.x

[24] Peters GL . Migraine overview and summary of current and emerging treatment options. Am J Manag Care 2019;25(Suppl 2):S23–S34. 30681821

